# Immunoglobulin G4-related periodontitis: case report and review of the literature

**DOI:** 10.1186/s12903-021-01592-2

**Published:** 2021-05-28

**Authors:** Jinmei Zhang, Lei Zhao, Jieyu Zhou, Wei Dong, Yafei Wu

**Affiliations:** 1 State Key Laboratory of Oral Diseases, West China College of Stomatology, Sichuan University, Chengdu, Sichuan China; 2 National Clinical Research Center for Oral Diseases, West China College of Stomatology, Sichuan University, Chengdu, Sichuan China; 3 Department of Periodontics, West China School and Hospital of Stomatology, Sichuan University, No. 14 Renim South Road 3rd Section, Chengdu, 610041 Sichuan China

**Keywords:** IgG4-related disease, Periodontitis, Diagnosis, Therapy, Differential diagnosis

## Abstract

**Background:**

Immunoglobulin G4-related disease (IgG4-RD) is a chronic inflammatory systemic disease of unknown etiology that can affect one or multiple organs. The disease can mimic many infectious and inflammatory diseases, mainly causing organ enlargement or hyperplasia. Its diagnosis primarily relies on clinical, serologic, and histological features (lymphoplasmacytic infiltrates, storiform fibrosis, and obliterative phlebitis of IgG4 + plasma cells). Here, we report a rare case of IgG4-related periodontitis, and review the relevant literatures.

**Case presentation:**

A 38-year-old Chinese man visited the Department of Periodontics with gingival enlargement, loose teeth, and tooth loss. The patient had very poor oral hygiene and a large amount of calculus. Gingivae were edematous with deep periodontal pockets and attachment loss. Panoramic radiography showed alveolar bone loss. Serologic examination showed that IgG was 23.70 g/L and IgG4 concentration was 2.800 g/L. There was significant lymphoplasmacytic infiltration, a storiform pattern of fibrosis, and mitotic figures with hematoxylin and eosin staining; immunohistochemical staining showed 10 scattered IgG4-positive plasma cells in a high-power field. The patient was diagnosed as IgG4-related periodontitis. He received a course of corticosteroids with periodontal therapy, and the enlargement was significantly improved without recurrence.

**Conclusion:**

IgG4-RD in the oral and maxillofacial region mainly involves salivary glands, but this rare case was characterized by enlarged gingivae. The differential diagnosis of IgG4-RD should be based on the clinical features and serologic (IgG4) and histopathological examinations. Corticosteroid therapy is effective for most IgG4-RD patients. Taken together, we hope this case report and the literature review can help dentists to improve their understanding of the IgG4-RD.

**Supplementary Information:**

The online version contains supplementary material available at 10.1186/s12903-021-01592-2.

## Background

Immunoglobulin G4-related disease (IgG4-RD) is a chronic inflammatory and systemic disease of unknown etiology that involves various organs, such as the pancreas, bile duct, orbital tissues, lacrimal and salivary glands, lungs, skin, liver, arteries, kidneys, retroperitoneum, prostate, gallbladder, and lymph nodes [1–4]. IgG4-related pancreatitis was first reported in 2001 [1, 2]. The incidence of IgG4-RD in Japan is reported to be 2.8 to 10.8 per million people and the prevalence is concentrated in middle-aged and older men [3, 4]. The clinical presentation of IgG-RD is varied and non-specific. Some patients are asymptomatic, while others present with organ dysfunction or masses, pseudotumoral enlargement, fibrosis, sclerosis, hyperplasia, or granuloma; clinical symptoms of some IgG4-RDs may be similar to those of malignant tumors [5–7]. Here, we report a rare case of IgG4-related periodontitis and review the relevant literatures on the clinical manifestations and pathological features of IgG4-RDs in the oral and maxillofacial region, and provide information on differential diagnosis, treatment, and prognosis of IgG4-RDs.

### Case presentation

A 38-year-old Chinese man visited the Department of Periodontics (West China Hospital of Stomatology, Sichuan University, Sichuan, China) with gingival enlargement, loose teeth, and tooth loss**.** The chief complaint of the patient was generalized gingival enlargement beginning 2 years ago, sometimes with pus. The teeth gradually loosened several months ago, and the left maxillary first molar was lost following severe pain. A local doctor diagnosed him as periodontitis, and then he was referred to the Department of Periodontics for periodontal therapy.

**Fig. 1:**
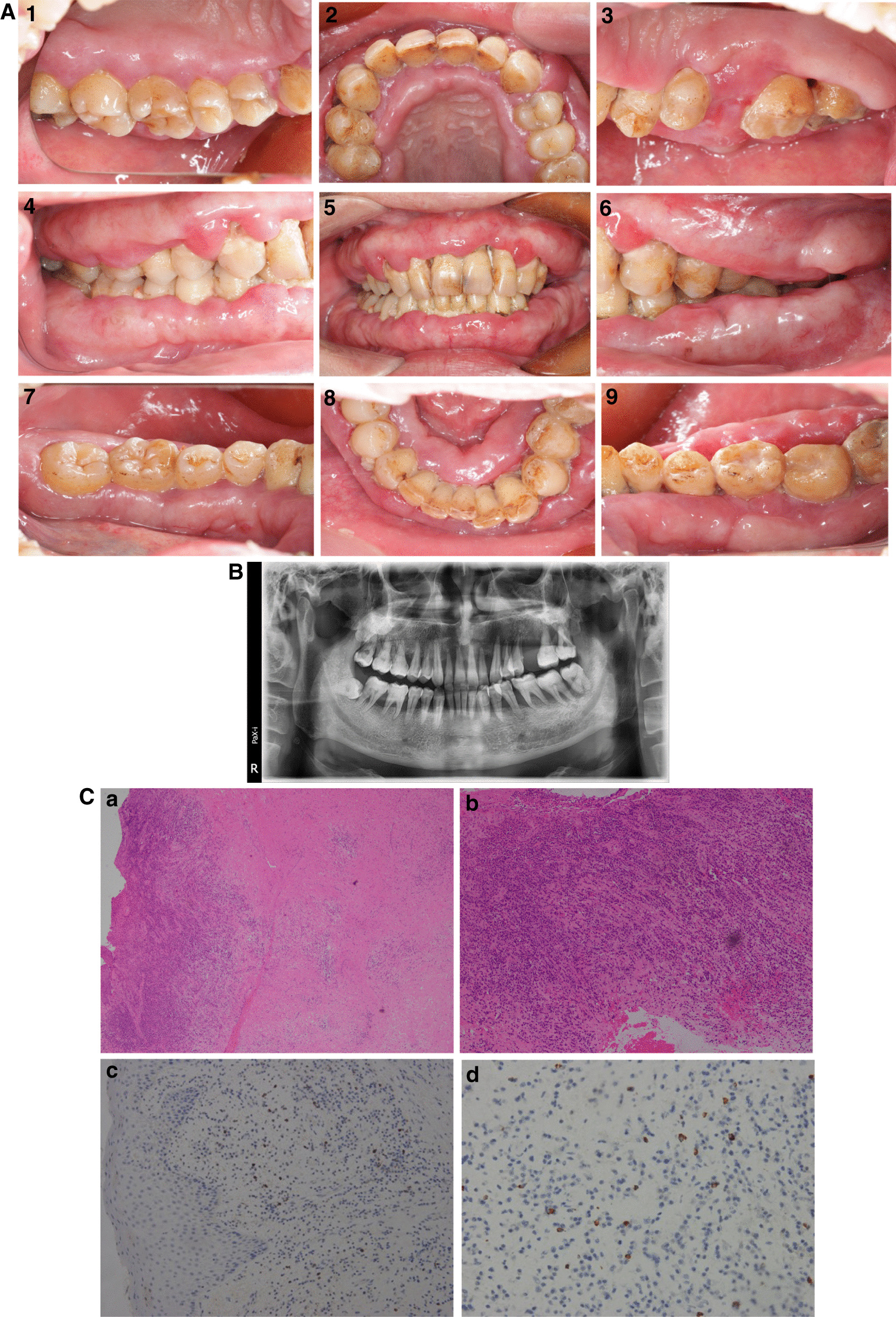
**A** Intraoral examination revealed generalized gingival swelling, calculus and poor oral hygiene, gingival margin was inflamed, gingivae were edematous and tenderness; **B** Panoramic radiograph showed alveolar bone resorption; **C a, b**: Histologic examination using hematoxylin-erosin (H&E) staining showed ulceration, a large number of lymphoplasmacytic cells infiltration and a storiform pattern of fibrosis, with occasional mitotic figures. (Magnification: a, ×40; b, ×100), arrowheads indicate a storiform pattern of fibrosis. **c, d**: Immunohistochemical staining was positive index for IgG4 (10/HPF) (magnification c ×200; d ×400), arrowheads indicate IgG4 + cells and a storiform pattern of fibrosis

Medical history: The patient was a smoker for 20 years, averaging 20+ cigarettes per day. There was no relevant family history and no special disease history, no history of allergies, and the patient was not on any drugs. The extraoral examination showed facial symmetry and no obvious abnormalities in the lymph nodes of the head and neck. Intraoral examination showed that the patient has extremely poor oral hygiene, with a mass of dental plaque and debris covering almost all the teeth. The teeth had subgingival and supragingival calculus from the incisors to the molars. Gingivae were generally enlarged and red, and the buccal gingivae of the left mandible and maxilla were even enlarged toward the occlusal surface. The involved gingivae were edematous and tender, the gingival margin was inflamed, and there was no ulceration or significant necrosis. General findings were bleeding on probing, deep periodontal pockets, and attachment loss. Tooth mobility varied from I^o^-III^o^, and was III^o^ in the left maxillary first premolar and second premolar (Fig. 1A). Mucosal texture and color were basically regular. There were no specific findings in the salivary glands, and salivary gland ducts could open normally. Panoramic radiography showed generalized alveolar bone loss (ABL), especially alveolar bone resorption at the left maxillary first and second premolars, as well as the left and right posterior teeth from alveolar crest to the apical area, but the maxillary sinus was intact without damage (Fig. 1B). No significant abnormalities were found in further clinical examinations, bone marrow examination, and tests for infectious diseases such as acquired immunodeficiency syndrome (AIDS), syphilis, hepatitis B and C, and mycobacterium tuberculosis. Serological examination showed that C-reactive protein was high at 14 mg/L (normal range: 0–10 mg/L), the serum IgG was 23.70 g/L (normal range: 8.00–15.50 g/L), and IgG4 concentration was 2.800 g/L (normal range: 0.035–1.500 g/L). Then biopsy of gingivae was carried out under anesthesia from the left maxillary first and second molar.

Histological analysis of hematoxylin and eosin staining (H&E) demonstrated ulceration, a large infiltration of lymphoplasmacytic cells, a storiform pattern of fibrosis, and mitotic figures (Fig. 1C-a, b); the preliminary diagnosis was plasma cell granuloma. Immunohistochemical (IHC) staining results showed that scattered infiltration of 10 IgG4-positive plasma cells in the high-power field (HPF) (Fig. 1C-c, d). Other IHC markers, CD38, Ki-67/mum, kappa, lambda, EBER, and Pan Cytokeratin (PCK) were positive (Additional file 1: Figure S1, data not shown). Position emission tomography-computed tomography (PET-CT) also excluded the possibility of cancer, and there were no obvious changes in other organs. According to clinical manifestations and serological and histological features, the lesion was regarded as an inflammatory condition. After 2 months, the patient was diagnosed as IgG4-related periodontitis by the Department of Rheumatology and Immunology (West China Hospital of Sichuan University) on the basis of the diagnostic criteria for IgG4-RD, although there were only 10 IgG4-positive plasma cells in the HPF. Using a new classification of periodontitis from a 2017 workshop [8–14], he was then diagnosed as periodontitis (Stage III, generalized, Grade C) and classified as manifestation of systemic diseases by Department of Periodontics.

**Fig. 2:**
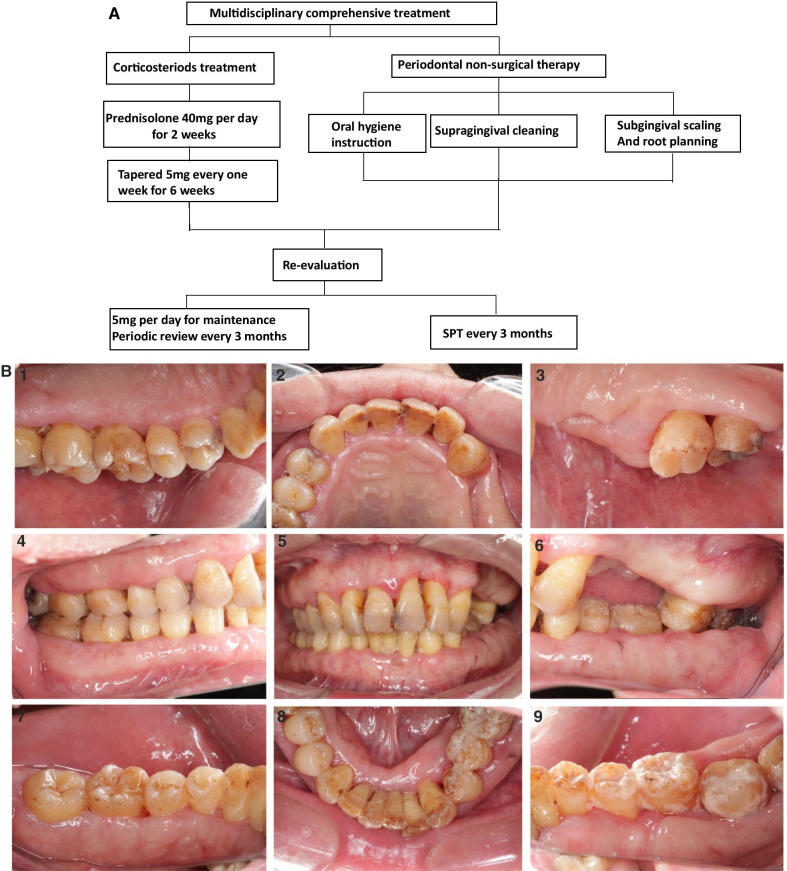
**A** Multidisciplinary comprehensive treatment approach with corticosteroids and periodontal non-surgical therapy; **B** After 6 months of multidisciplinary comprehensive treatment, the swelling of the gingivae significantly improved, the gingival edema disappeared and the gingivae recessed

The treatment plan for this patient was oral corticosteroids and periodontal therapy guided by the Department of Rheumatology and Immunology (West China Hospital of Sichuan University) and the Department of Periodontics. The rheumatologist recommended oral corticosteroids therapy. The initial dose of prednisolone was 0.6 mg/kg for 2 weeks (40 mg/day), which was then tapered by 5 mg every week for 6 weeks to determine a maintenance dose (5 mg/day). At the same time, the periodontist carried out periodontal therapy, including oral hygiene instruction, supragingival cleaning, subgingival scaling, and root planning (Fig. 2A). Three months later after non-surgical therapy, the periodontist re-evaluated the periodontal condition of the patient, then suggested he should have supportive periodontal therapy (SPT).

After 6 months of multidisciplinary comprehensive treatment, an approach using corticosteroids with periodontal non-surgical therapy, gingival edema disappeared, the color of the gingivae turned pink, gingivae recessed, periodontal pockets became shallow, and gingival enlargement significantly decreased (Fig. 2B). In serological testing, IgG4 concentration was 0.584 g/L, which was in the normal range. After this, the rheumatologist recommended a supportive dose of 5 mg/day and revisit every 3 months, while the periodontist suggested that patient should have SPT every 3 months and quit smoking (timeline in Additional file 2: Figure S2, data not shown).

## Discussion and conclusions

IgG4-RD is a fibroinflammatory disease characterized by diffuse/localized enlargement in one or multiple organs, elevated serum IgG4 levels, and extensive infiltration of IgG4-positive plasma cells [15–18]. Although the etiology of IgG4-RD is not clear, it is possibly caused by a genetic, microbiological, or persistent antigenic or autoimmune stimulus [19]. In addition, several studies demonstrated that IgG4-RD is a Th2-dependent disease [1, 20], and it can be a result of immunological and allergic conditions in different organs [19]. IgG4-RDs include autoimmune pancreatitis, Mikulicz disease (MD), pachymeningitis, hypophysitis, orbital pseudotumor, chronic sclerosing dacryoadenitis, pericarditis, sclerosing cholangitis, prostatitis, and other disorders [2, 21, 22]. IgG4-RDs occur most commonly in the pancreas, followed by the head and neck regions (particularly in the salivary glands), then skin, orbit, lymph nodes, thyroid, upper airway, and respiratory tract [23, 24]. IgG4-RDs may be responsible in patients with hyperplasia, enlargement, compression, obstruction, or organ dysfunction in these areas [6, 7]. What is more, another characteristic feature of IgG4-RD is the possibility of recurrence [1]. Based on the consensus [25], there are three comprehensive diagnostic criteria for IgG4-RD: (1) characteristic diffuse/localized enlargement or masses in single organ or multiple organs, (2) elevated serum IgG4 concentrations, and (3) dense lymphoplasmacytic infiltrates, storiform fibrosis, and obliterative phlebitis with a significant number of IgG4 + plasma cells: IgG4 + /IgG > 40% or > 10 IgG4 + plasma cells/high-power field (HPF). Under the standard diagnostic criteria, the finding of all three means definite disease, the finding of (1) and (2) means probable, and (1) and (3) means possible.

In this case, the clinical sign was generalized gingival enlargement, involving both free and attached gingivae, and serologic examination showed the serum IgG4 concentration was 2.800 g/L (normal range: 0.035–1.500 g/L). In addition, the histological examination (H&E and IHC) showed marked lymphoplasmacytic infiltration and fibrosis, with scattered infiltration of 10 IgG4-positive plasma cells in the HPF, so the final diagnosis in this patient was IgG4-related periodontitis. Although there was no predominant venulitis, it is possible that due to the size and location of the lesions, restricted sampling obscured venulitis [26]. Everds NE et al. reported that pre-analytical and analytical variables and related factors, for example, sample fixation could have an impact on clinical pathology [27].

There are many reasons for gingival enlargement, such as dental plaque, drugs, autoimmune response, and genetic factors, as well as systemic diseases. Local and generalized gingival enlargement caused by IgG4-RDs is similar to plasma cell granuloma, drug-induced gingival enlargement, hereditary gingival fibromatosis, epulis, periodontitis, and leukemia (Table 1). One study showed that plasma cell granuloma can also be accompanied by high serum concentration of IgG4, a mean number of IgG4 + plasma cells (HPF) more than 10, and an IgG4 + /IgG plasma cell ratio more than 40%, but it is not an IgG4-RD [6] because there is no lymphoplasmacytic infiltrate, storiform fibrosis, or obliterative phlebitis in plasma cell granuloma. Because of the similarity, the definitive diagnosis of IgG4-RD needs to have comprehensive diagnostic criteria. Therefore, oral diseases involved with IgG4-RDs should be differentially diagnosed through clinical features, medical history, radiology studies, and histopathological examination to avoid misdiagnosis and delaying diagnosis.

**Table 1:** Clinicopathological features and differential diagnosis of common gingival swelling

Disease	Etiology	Clinical features	Histopathological features
Plasma cell granuloma [6, 28]	Allergenic factors: food, toothpaste, tobacco	Short duration, intensely erythematous gingiva and/or ulceration	A large infiltration of plasma cells
Drug-induced gingival Enlargements [29]	Dental plaque, drugs including Phenytoin, Cyclosporine, Nifedipine, Verapamil	Long-term medication history, generalized pale and pink gingivae, tough texture, slightly elastic, hard to bleed	Significant thickened stratum spinosum
Hereditary gingival Fibromatosis [30]	Mutation localized to 2p21-p22&5q13-q22; Mutation of “Son of Sevenless” genes [31, 32]	Generalized fibrous gingival enlargement of tuberosities, anterior free/attached gingiva retro-molar pads	Significant thickened stratum spinosum, a large number of fibroblasts
Epulis [33]	Dental plaque; hormone; Trauma	Local swelling, bleeding, recurrence	Fibroblasts, granulomatous cells
Periodontitis [11, 14]	Dental plaque, heredity, age, gender, smoking	Deep pocket, periodontal attachment loss	Various inflammatory cells
Gingival swelling with leukemia [34]	Immunosuppression due to malignant transformation of leukocyte production in the bone marrow	Gingival swelling and bleeding due to leukemic cell infiltration. Ulceration and necrosis on gingiva and tooth mobility	Primarily undifferentiated leukocytes

In the oral and maxillofacial regions, the occurrence of involved gingivae in IgG4-RDs (especially IgG4-related periodontitis) is relatively rare, and the most common diseases are MD and Kuttner’s tumor (also known as chronic sclerosing dacryoadenitis) [15]. Patients with MD develop bilateral, symmetrical, painless enlargement of their lacrimal and salivary (parotid and submandibular) glands. CT usually shows the lacrimal gland, parotid gland, and submandibular gland enlargement. Serological and pathological findings are consistent with IgG4-RDs [15, 16, 35]. In addition to MD, Kuttner’s tumor is thought to be another common lesion of IgG4-RDs in the oral and maxillofacial regions. The clinical manifestations are similar to those of other tumors, but Kuttner’s tumor is usually benign, occurring with an immune disorder. The main clinical manifestations are hard, painless masses in the salivary glands, and radiological examination can show disappearance of the acinus and ductal dilatation. The histological manifestation is ductal fibrosis of the salivary gland, with significant connective tissue proliferation and hyaline degeneration; acinar atrophy disappears, replaced by a large number of lymphocytes and plasma cells [35]. These two diseases are especially easy to confuse with Sjögren's syndrome (SS), and sometimes the early symptoms of MD and Kuttner’s tumor will require differentiation from SS. However, the biggest difference is that anti-SSA (anti-Ro) antibodies and anti-SSB (anti-La) antibodies can be detected in SS patients [15].

We performed a literature review through the PubMed database using the terms “IgG4-related,” “case,” “oral,” and “maxillofacial” [4, 7, 35–49]. We found that the main sites of IgG4-RDs in the oral and maxillofacial region are salivary glands (11 cases), lymph nodes (3 cases), and maxillary sinus (7 cases). Beyond that, sites included alveolar mucosa (3 cases), hard palate (2 cases), floor of mouth (1 case), and facial nerve (2 cases) (Table 2). Gingivae, as in this case, were rarely reported. In addition, more than one site was involved in 3 cases. Among all the reported cases, the ratio of males/females was 17:6, and the average age was around 61.13 years old. The number of IgG4 + /HPF was as high as 403, and the highest ratio of IgG4 + /IgG was 94% (Table 2). Because the clinical features are asymptomatic and nonspecific, and the sensitivity and specificity of serologic examination is only 60% [50], it is mandatory to do a biopsy for diagnosis of IgG4-RDs. Although IgG4 concentration surprisingly reached 30.31 g/L in a previous case, it is worth noting that the IgG4 level can also be elevated in specific and non-specific inflammation, autoimmune diseases (systemic lupus erythematosus, SS, and vasculitis), other tumors, pancreatic cancer, bile duct cancer, primary immunodeficiency disease, and interstitial pneumonia [51]. It is also worth mentioning that there were 5 cases diagnosed as IgG4-RD with normal serum IgG4 level, which indicated that the serum IgG4 index has limitations for diagnosis of IgG4-RDs. However, if there is an abnormal IgG4 serologic finding, diseases related to high IgG4 concentration such as IgG4-RDs should be considered.

**Table 2:** The IgG4 + plasma cells/HPF and IgG4 + /IgG ratio of reported IgG4-RD in oral and maxillofacial region

Sites	Sex/age	IgG4 + /HPF	IgG4 + /IgG (ratio)	Serum IgG4 concentration (g/L)	References
Submandibular gland	Female/66	N/D	N/D	142.7	Bukhari [4]
	Male/56	N/D	N/D	2.86	Anand [35]
	Male/60	N/D	47%	3.14	Akiyama [36]
	Male/46	N/D	> 70%	Normal	Gontarz [37]
	Male/53	N/D	> 50%	3.27	Sun [38]
	Female/64	N/D	> 40%	Normal	Tanaka [39]
	Male/77	63	50%	4.25	Abe [40]
	Male/62	403	94%	6.68	Abe [40]
Parotid gland	Female/66	N/D	N/D	142.7	Bukhari [4]
	Female/71	280	80%	30.31	Andrew [41]
	Male/73	diffuse	75%	Normal	Ishida [42]
Lymph nodes	Male/46	N/D	> 70%	Normal	Gontarz [37]
	Male/30	N/D	80%	3.35	Gontarz [37]
	Male/63	N/D	> 40%	4.66	Wu [43]
Maxillary sinus	Male/48	135	40%	Normal	Kouwenberg [44]
	Male/30	75	85%	3.35	Gontarz [37]
	Male/67	N/D	N/D	2.43	Kojima [45]
	Male/62	N/D	N/D	9.1	Kojima [45]
	Male/50	39	77%	2.58	Ikeda [46]
	Male/73	N/D	20%	Normal	Pace [47]
	Male/73	diffuse	72%	Normal	Ishida [42]
Lower alveolar Mucosa	Male/79	139	72%	1.65	Laco [7]
Upper alveolar	Male/74	66	71%	N/D	Laco [7]
Mucosa	Male/30	75	85%	3.35	Gontarz [37]
Hard Palate	Female/71	280	80%	30.31	Andrew [41]
	Female/66	N/D	N/D	142.7	Bukhari [4]
Floor of mouth	Female/59	103	68%	1.85	Laco [7]
Facial nerve	Female/61	> 50	N/D	Normal	Wick [48]
	Female/74	279	25%	N/D	Yuichi Segawa [49]
Upper gingiva	Male/38	10	N/D	2.8	This case
Lower gingiva	Male/38	10	N/D	2.8	This case

*HPF*: high power field

*N/D* not describe

Overall, the common chief complaints of patients are neck enlargement, dry mouth, enlargement of lymph nodes, gingival hyperplasia, and mucosal enlargement. The clinical manifestations in the oral and maxillofacial regions include enlargement and painless hard masses in the salivary glands, and the radiographs often show enlargement of the salivary glands. Even though some IgG4-RDs appear first in the oral cavity, the symptoms in other organs can’t be overlooked, since 7 previous cases demonstrated other organs involvement (Table 3). Among 23 cases, five cases were initially misdiagnosed and only correctly diagnosed after 7–20 months [7, 39, 42]. Xue et al. [20] reported a case of a 60-year-old woman who had been diagnosed as IgG4-RD after 19 years of misdiagnosis. In this period, the patient suffered multi-organ progressive enlargement, involving the parotid glands, lacrimal glands, kidneys, submandibular glands, salivary glands, pituitary, pancreas, and lung. Because of the misdiagnosis, the patient was admitted to the hospital frequently. Additionally, another patient’s condition was exacerbated 7 months after surgery because of misdiagnosis, until the treatment plan was adjusted to include corticosteroids therapy, the serum IgG4 level significantly decreased and the symptoms were relieved [39]. This is a good illustration that dentists should also pay attention to patients’ systemic diseases in addition to oral diseases.

**Table 3:** The clinical manifestation, treatment, progression and prognosis of reported IgG4-RD in oral and maxillofacial region

Case	Sex/age	Site	Clinical manifestation	Radiogragh	Pathological feature	Other organ involvement
Bukhari AF	Female/66	Hard palate SG Parotid gland	Swelling, a boggy consistency and red–purple discoloration	Swelling of SG	Atypical proliferation of lymphocytes and plasma cell	Chest, abdominal pancreas, lung
Kouwenberg	Male/48	Left maxillary alveolar bone maxillary sinus	Hypermobile teeth necrosis of the left maxillary alveolar process	Destruction of left maxillary alveolar bone and pattern maxillary sinus	Numerous plasma cells fibrosis in a storiform	N/D
Gontarz	Male/30	cervical lymph nodes upper alveolar mucosa maxillary sinus	Enlargement of cervical lymph nodes, inflammatory granulation tissues, loosening of tooth	A defect of the alveolar part of the left maxilla	Lymphocytes, plasma cells, many eosinophils	N/D
Gontarz	Male/46	SG	Cirrhosis of SG	Normal	Dense lymphoplasmacytic Infiltrate, stromal fibrosis	N/D
Kojima	Male/62	Maxillary sinus	Facial swelling	Bilateral consolidation with a peribronchial distribution	Lymphocytes, plasma cells, scattered eosinophils	N/D
Kojima	Male/67	Maxillary sinus	Cough, sputum right nasal obstruction	Diffuse bilateral thickening of the mucosa of the maxillary, frontal sinuses	Lymphocytes, plasma cells, scattered eosinophils	N/D
Anand K	Male/56	SG	Dry mouth sensation, neck swelling	Enlargement of SGs	A dense lymphoplasmacytic infiltrate germinal center	N/D
Andrew	Male/56	Hard palate Parotid gland	Erythematous, nontender, bilateral nodules	Enlargement of parotid glands	N/D	N/D
Laco	Female/59	Floor of mouth	Asymptomatic swelling	N/D	Obliterative phlebitis, lymphoid	N/D
Laco	Male/79	Lower left alveolar mucosa	Asymptomatic swelling	N/D	Obliterative phlebitis	N/D
Laco	Male/74	Upper left alveolar mucosa	Asymptomatic swelling	N/D	N/D	N/D
Sun	Male/53	SG	Painless, hard left neck mass, slight dysfunction of salivary secretion	Left swollen SG	Atrophy, abundant lymphocytoplasma cells	Pancreas
Tanaka	Female/64	SG	Swelling of the right submandibular region	Enlargement of the right SG and atrophy of the left SG with a salivary stone	A ratio of IgG4/IgG-positive plasma cells of more than 50%	Kidney and pancreas
Akiyama	Male/60	SG	Swelling of bilateral SGs	Enlarged bilateral SGs	Heavy infiltration of lymphocytes and hyperplastic germinal center formation	Kidney
Abe	Male/77	SG	Bilateral SG swelling	Diffused enlargement of the bilateral SGs	Diffuse infiltration of lymphocytes and plasma cells and more severe periductal fibrosis	Right nasal septum
Abe	Male/62	SG	Swelling hard mass of submandibular region	Diffused enlargement of SGs	Cellular infiltration with lymphocytes, plasma cells, and eosinophils around the ducts	N/D
Ishida	Male/73	Right maxillary sinus parotid gland	Tumor	A tumor with destruction of the surrounding bone tissues in the maxillary sinus	Hyalinized dense fibrosis in the right maxillary sinus and parotid gland	N/D
Ikeda	Female/50	Maxillary sinus	N/D	A soft shadow in the maxillary sinus	Submucosal infiltration of a number of lymphocytes and plasma cells with fibrosis	N/D
Pace	Male/73	Maxillary sinus	Facial swelling	Opacification of the right maxillary sinus and bone destruction	Inflamed collagenous connective tissue, foci of cholesterol crystals	N/D
Wu	Male/63	Cervical lymph nude	Enlarge masses over bilateral posterior neck	Enlarged lymph nodes	Scattered plasma cells and focal penetration of blood vessels in germinal centers	N/D
Wick	Female/61	Facial nerve	Cranial nerve VI and VII palsies	Homogenous enhancing mass	Plasmacytic infiltration, storiform, fibrosis, and phlebitis	Ear
This case	Male/38	Upper and lower gingiva	Swelling of ginigva	Alveolar bone loss	Lymphoplasmacytic infiltration and a storiform pattern of fibrosis	N/D

*SG* submandibular gland

*N/D* not describe

Currently, there is no standard treatment plan for IgG4-RDs, but they are generally sensitive to corticosteroids therapy. The recommended dosage is prednisone 0.6 mg /kg/day for 2 to 4 weeks. After 3–6 months, it is gradually reduced to 5 mg/day, and then 2.5–5 mg/day for 3 years [37, 40, 43, 44, 48]. The dosage of corticosteroids can be adjusted according to the severity of the patient’s condition. In one case, the patient was given pulsed therapy with 200 mg of methylprednisolone and 20 g gamma globulin via intravenous infusion [20]. After corticosteroid therapy, the prognosis is good, with a return to a normal serum IgG4 level and no recurrence. Patients often maintain a 5 mg/day dosage under guidance of a physician for several months or years, and the probability of recurrence is quite low. In addition to corticosteroid therapy, there is also rituximab therapy for patients who are steroid resistant [41, 52].

This review reported a rare clinical case of IgG4-related periodontitis with generalized and severe gingival enlargement in the oral and maxillofacial region. As systemic diseases, IgG4-RDs have gradually gained the attention of medical community but the etiology and pathology of these diseases are still unclear. The clinical features of IgG4-RDs are diverse and non-specific, involving multiple organs, and diagnosis should be based on the clinical, serologic, and histological features (ratio of IgG4/IgG or number of plasma cells in HPF); otherwise, IgG4-RDs are easily misdiagnosed. In addition, an early and correct diagnosis is the main goal for the future studies. When we see abnormal edematous and hyperplastic gingivae that are different from the regular periodontal manifestations, serologic examinations should be performed first. If the serology is normal, we should consider biopsy and immunohistochemical staining of local tissues. The combination of serologic, histological, and clinical features can be a clue for further investigation. Of course, periodontitis as a rare manifestation of systemic disease is indeed confusing and difficult to diagnose.

At present, several researchers are dedicated to developing more optimal, specific classification standards that can help to diagnose IgG4-RDs more quickly and accurately, but the relationship between IgG4-RDs and periodontitis still needs further investigation. Additionally, many oral symptoms may be associated with systemic diseases, so it is essential to pay attention to oral health. In conclusion, improvements are needed in correctly diagnosing and treating IgG4-RD, avoiding misdiagnosis, delivering timely treatment, alleviating symptoms, and improving prognosis.

## Supplementary Information

**Additional file 1**. Immunohistochemical staining was positive index for CD38 (a, b), Ki-67/mib-1(c, d), mum1(e, f), kappa (g, h), lambda (i, j), EBER (k, l), PCK (m, n). (magnification a, c, e, g, i, k, m ×200; b, d, f, h, j, l, n ×400)

**Additional file 2**. Timeline of treatment

## Data Availability

The datasets used and/or analyzed during the current study are available from the corresponding author on reasonable request.

## Abbreviations

IgG4-RD: Immunoglobulin G4-related disease
ABL: Alveolar bone loss
AIDS: Acquired immunodeficiency syndrome
H&E: Hematoxylin and eosin staining
IHC: Immunohistochemical staining
HPF: High-power field
PCK: Pan Cytokeratin
PET-CT: Position emission tomography-computed tomography
SPT: Supportive periodontal therapy
MD: Mikulicz disease
SS: Sjogren's syndrome
