# Supplementary figures and images for: Immunoglobulin G4-related periodontitis: case report and review of the literature
Source: BMC Oral Health. 2021 May 28;21:279. doi: 10.1186/s12903-021-01592-2 (PMC8161922; doi:10.1186/s12903-021-01592-2)

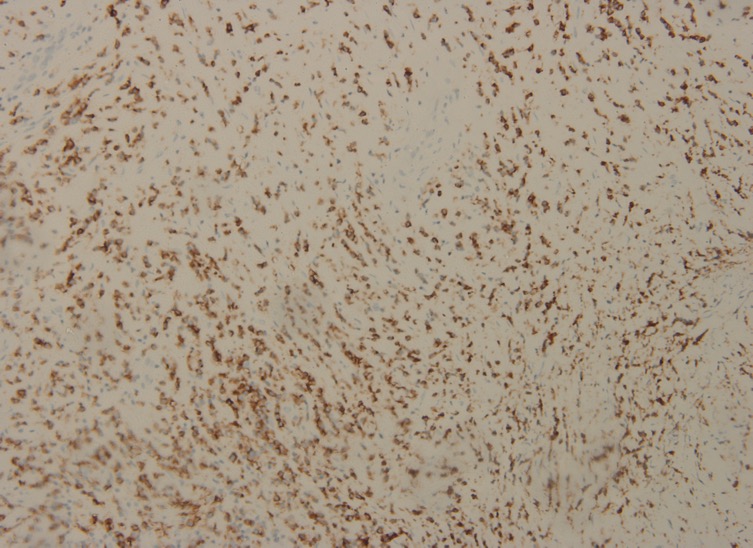

Supplement: Supplementary file 1 — Additional file 1. Immunohistochemical staining was positive index for CD38 (a, b), Ki-67/mib-1(c, d), mum1(e, f), kappa (g, h), lambda (i, j), EBER (k, l), PCK (m, n). (magnification a, c, e, g, i, k, m ×200; b, d, f, h, j, l, n ×400) [file 12903_2021_1592_MOESM1_ESM.zip › 12903_2021_1592_MOESM1_ESM/Figure S1-aR4.jpg]

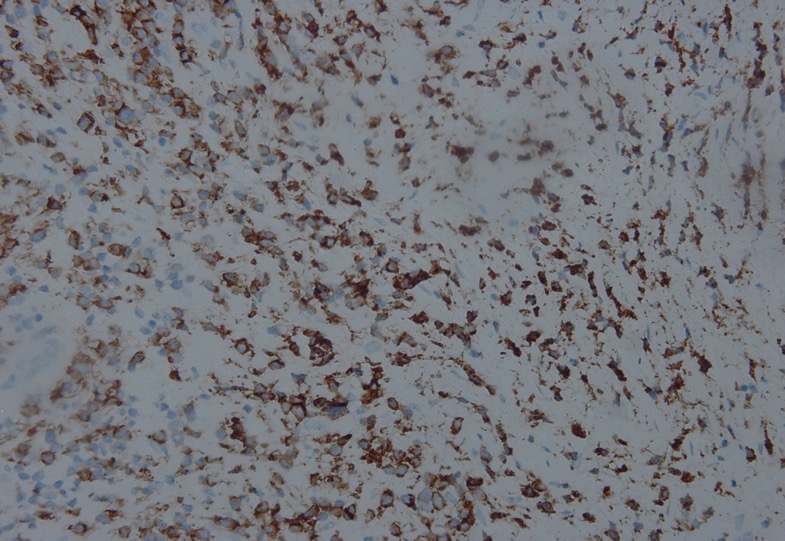

Supplement: Supplementary file 1 — Additional file 1. Immunohistochemical staining was positive index for CD38 (a, b), Ki-67/mib-1(c, d), mum1(e, f), kappa (g, h), lambda (i, j), EBER (k, l), PCK (m, n). (magnification a, c, e, g, i, k, m ×200; b, d, f, h, j, l, n ×400) [file 12903_2021_1592_MOESM1_ESM.zip › 12903_2021_1592_MOESM1_ESM/Figure S1-bR4.jpg]

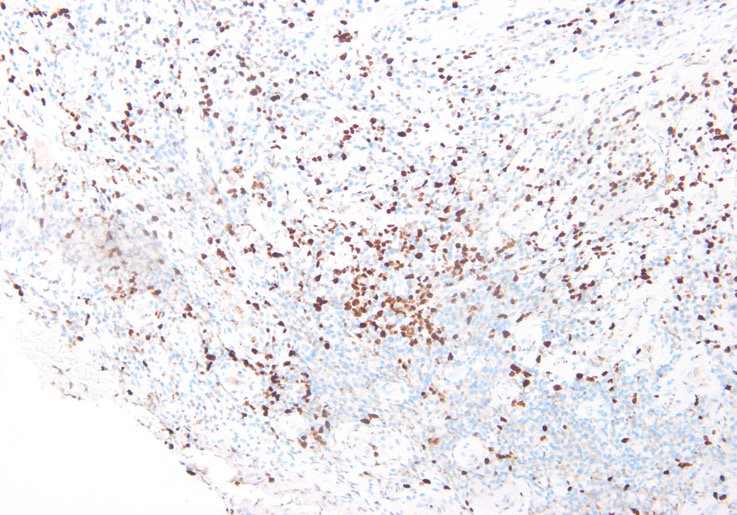

Supplement: Supplementary file 1 — Additional file 1. Immunohistochemical staining was positive index for CD38 (a, b), Ki-67/mib-1(c, d), mum1(e, f), kappa (g, h), lambda (i, j), EBER (k, l), PCK (m, n). (magnification a, c, e, g, i, k, m ×200; b, d, f, h, j, l, n ×400) [file 12903_2021_1592_MOESM1_ESM.zip › 12903_2021_1592_MOESM1_ESM/figure S1-cR4.jpg]

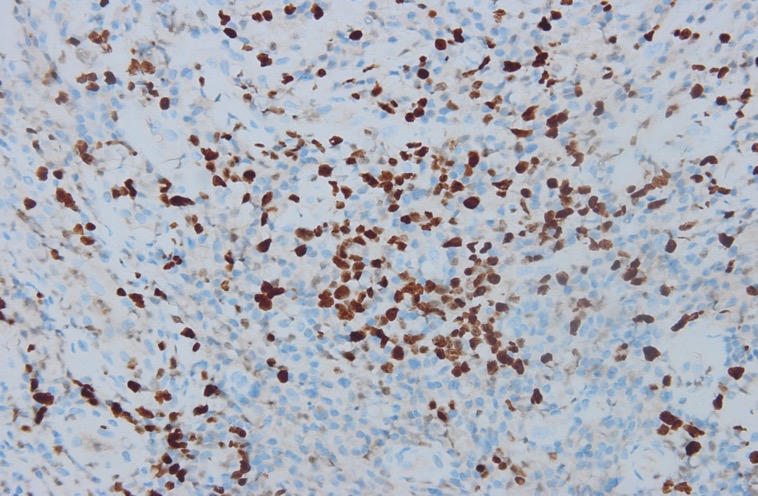

Supplement: Supplementary file 1 — Additional file 1. Immunohistochemical staining was positive index for CD38 (a, b), Ki-67/mib-1(c, d), mum1(e, f), kappa (g, h), lambda (i, j), EBER (k, l), PCK (m, n). (magnification a, c, e, g, i, k, m ×200; b, d, f, h, j, l, n ×400) [file 12903_2021_1592_MOESM1_ESM.zip › 12903_2021_1592_MOESM1_ESM/figure S1-dR4.jpg]

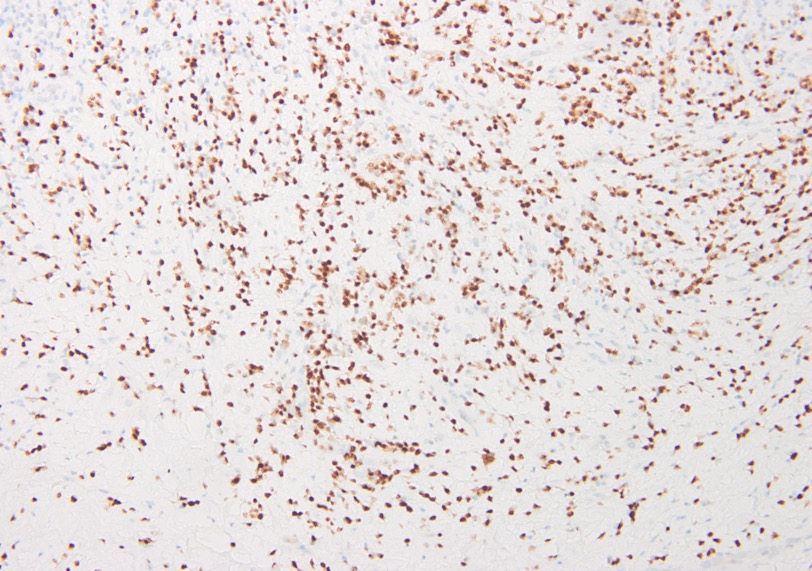

Supplement: Supplementary file 1 — Additional file 1. Immunohistochemical staining was positive index for CD38 (a, b), Ki-67/mib-1(c, d), mum1(e, f), kappa (g, h), lambda (i, j), EBER (k, l), PCK (m, n). (magnification a, c, e, g, i, k, m ×200; b, d, f, h, j, l, n ×400) [file 12903_2021_1592_MOESM1_ESM.zip › 12903_2021_1592_MOESM1_ESM/figure S1-eR4.jpg]

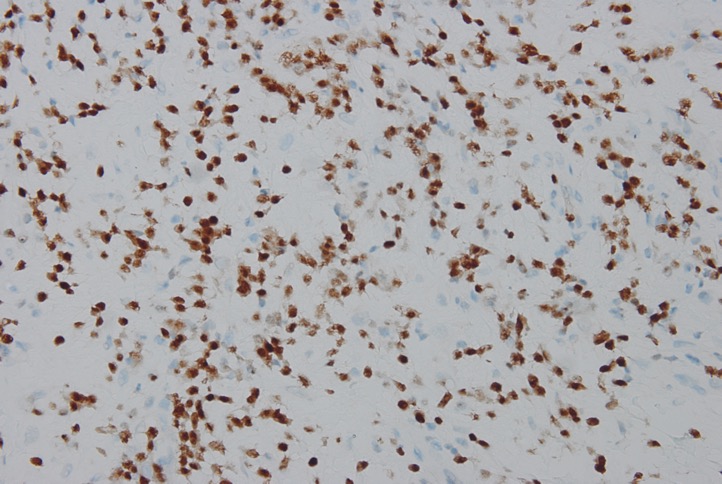

Supplement: Supplementary file 1 — Additional file 1. Immunohistochemical staining was positive index for CD38 (a, b), Ki-67/mib-1(c, d), mum1(e, f), kappa (g, h), lambda (i, j), EBER (k, l), PCK (m, n). (magnification a, c, e, g, i, k, m ×200; b, d, f, h, j, l, n ×400) [file 12903_2021_1592_MOESM1_ESM.zip › 12903_2021_1592_MOESM1_ESM/figure S1-fR4.jpg]

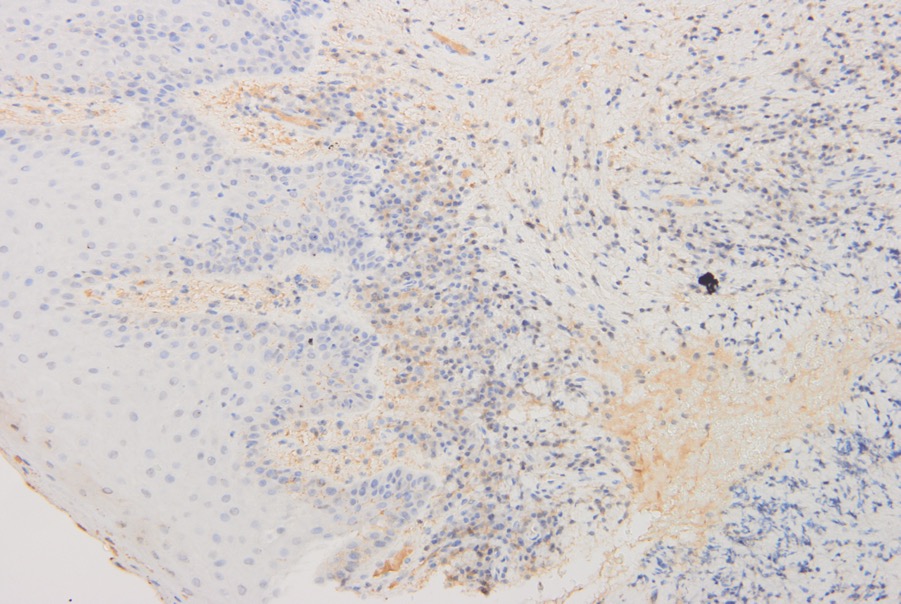

Supplement: Supplementary file 1 — Additional file 1. Immunohistochemical staining was positive index for CD38 (a, b), Ki-67/mib-1(c, d), mum1(e, f), kappa (g, h), lambda (i, j), EBER (k, l), PCK (m, n). (magnification a, c, e, g, i, k, m ×200; b, d, f, h, j, l, n ×400) [file 12903_2021_1592_MOESM1_ESM.zip › 12903_2021_1592_MOESM1_ESM/figure S1-gR4.jpg]

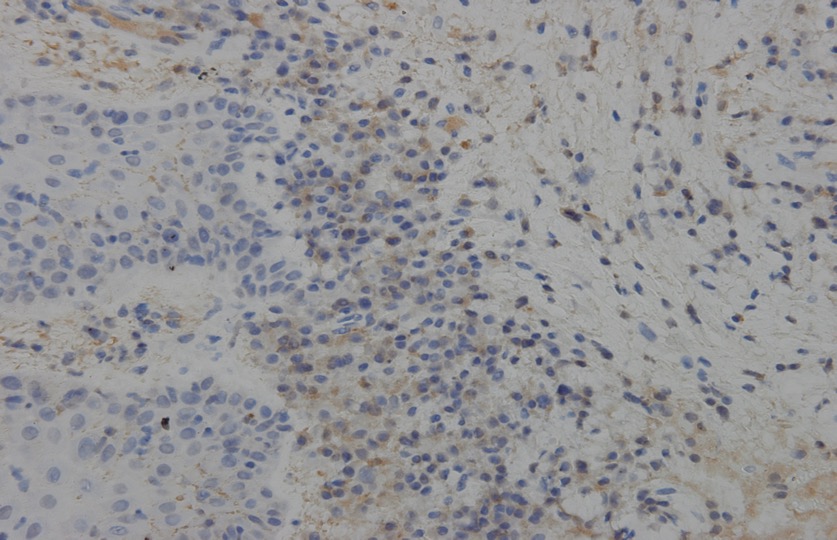

Supplement: Supplementary file 1 — Additional file 1. Immunohistochemical staining was positive index for CD38 (a, b), Ki-67/mib-1(c, d), mum1(e, f), kappa (g, h), lambda (i, j), EBER (k, l), PCK (m, n). (magnification a, c, e, g, i, k, m ×200; b, d, f, h, j, l, n ×400) [file 12903_2021_1592_MOESM1_ESM.zip › 12903_2021_1592_MOESM1_ESM/figure S1-hR4.jpg]

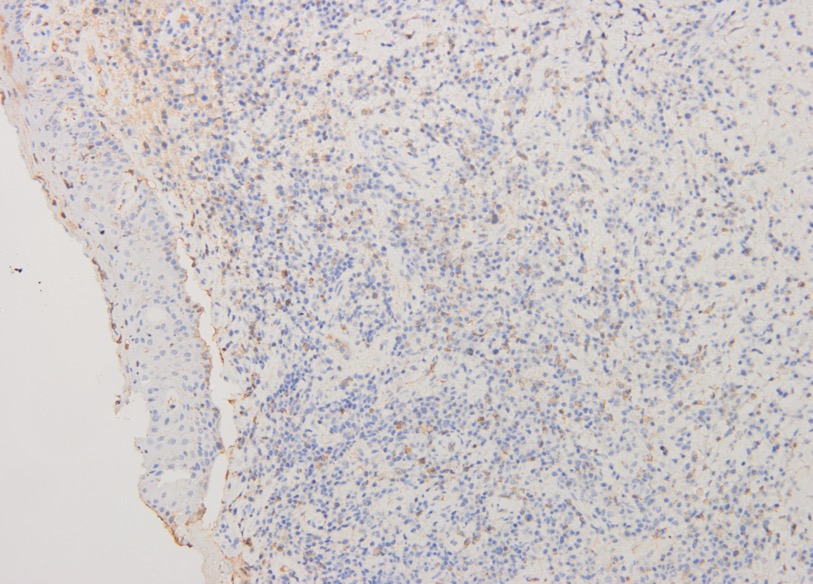

Supplement: Supplementary file 1 — Additional file 1. Immunohistochemical staining was positive index for CD38 (a, b), Ki-67/mib-1(c, d), mum1(e, f), kappa (g, h), lambda (i, j), EBER (k, l), PCK (m, n). (magnification a, c, e, g, i, k, m ×200; b, d, f, h, j, l, n ×400) [file 12903_2021_1592_MOESM1_ESM.zip › 12903_2021_1592_MOESM1_ESM/figure S1-iR4.jpg]

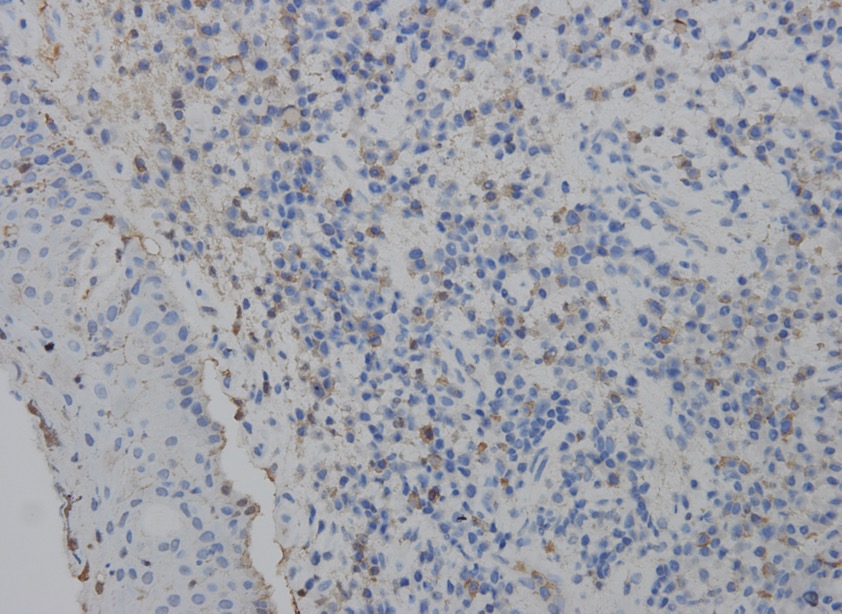

Supplement: Supplementary file 1 — Additional file 1. Immunohistochemical staining was positive index for CD38 (a, b), Ki-67/mib-1(c, d), mum1(e, f), kappa (g, h), lambda (i, j), EBER (k, l), PCK (m, n). (magnification a, c, e, g, i, k, m ×200; b, d, f, h, j, l, n ×400) [file 12903_2021_1592_MOESM1_ESM.zip › 12903_2021_1592_MOESM1_ESM/figure S1-jR4.jpg]

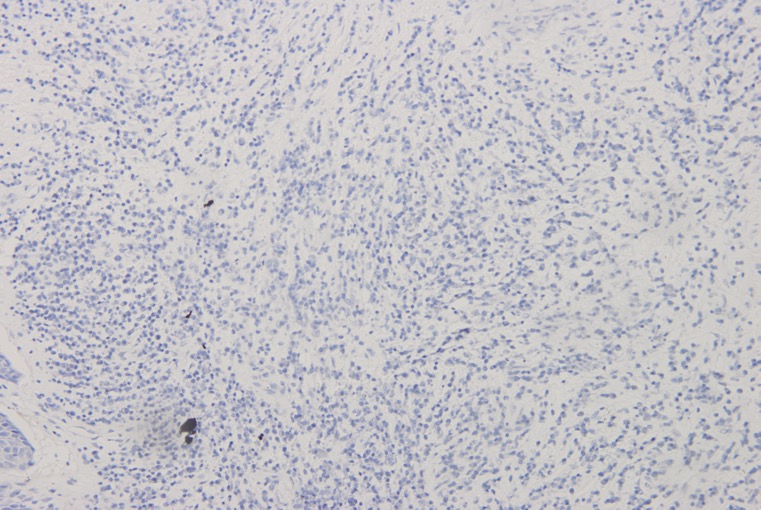

Supplement: Supplementary file 1 — Additional file 1. Immunohistochemical staining was positive index for CD38 (a, b), Ki-67/mib-1(c, d), mum1(e, f), kappa (g, h), lambda (i, j), EBER (k, l), PCK (m, n). (magnification a, c, e, g, i, k, m ×200; b, d, f, h, j, l, n ×400) [file 12903_2021_1592_MOESM1_ESM.zip › 12903_2021_1592_MOESM1_ESM/figure S1-kR4.jpg]

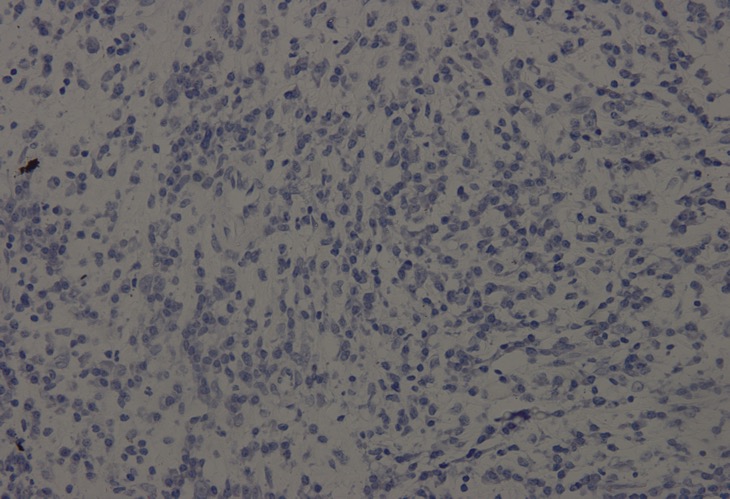

Supplement: Supplementary file 1 — Additional file 1. Immunohistochemical staining was positive index for CD38 (a, b), Ki-67/mib-1(c, d), mum1(e, f), kappa (g, h), lambda (i, j), EBER (k, l), PCK (m, n). (magnification a, c, e, g, i, k, m ×200; b, d, f, h, j, l, n ×400) [file 12903_2021_1592_MOESM1_ESM.zip › 12903_2021_1592_MOESM1_ESM/figure S1-lR4.jpg]

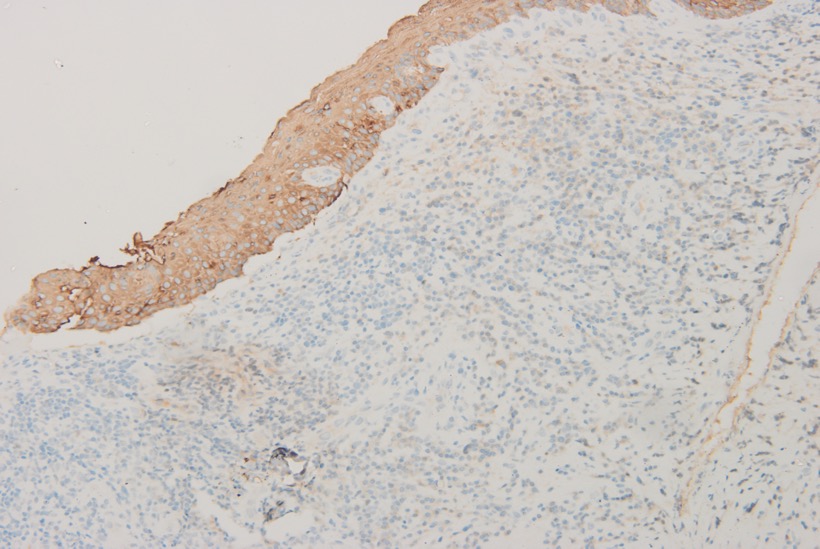

Supplement: Supplementary file 1 — Additional file 1. Immunohistochemical staining was positive index for CD38 (a, b), Ki-67/mib-1(c, d), mum1(e, f), kappa (g, h), lambda (i, j), EBER (k, l), PCK (m, n). (magnification a, c, e, g, i, k, m ×200; b, d, f, h, j, l, n ×400) [file 12903_2021_1592_MOESM1_ESM.zip › 12903_2021_1592_MOESM1_ESM/figure S1-mR4.jpg]

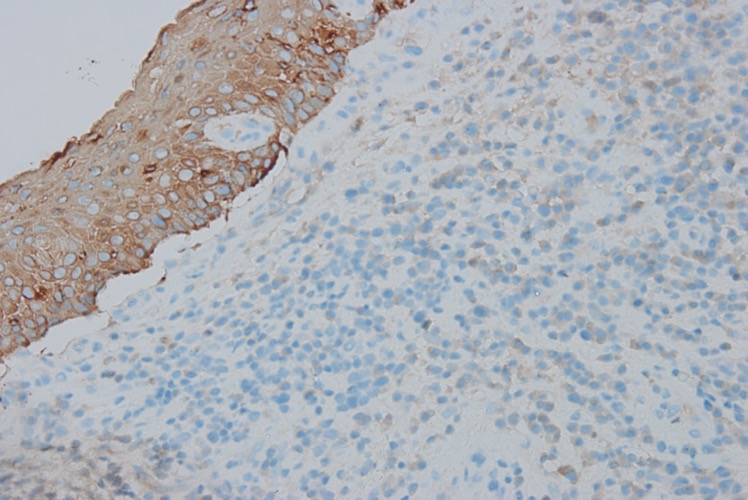

Supplement: Supplementary file 1 — Additional file 1. Immunohistochemical staining was positive index for CD38 (a, b), Ki-67/mib-1(c, d), mum1(e, f), kappa (g, h), lambda (i, j), EBER (k, l), PCK (m, n). (magnification a, c, e, g, i, k, m ×200; b, d, f, h, j, l, n ×400) [file 12903_2021_1592_MOESM1_ESM.zip › 12903_2021_1592_MOESM1_ESM/figure S1-nR4.jpg]
