# Supplementary material for: Immunoglobulin G4-related periodontitis: case report and review of the literature
Source: BMC Oral Health. 2021 May 28;21:279. doi: 10.1186/s12903-021-01592-2 (PMC8161922; doi:10.1186/s12903-021-01592-2)
Supplement: Supplementary file 2 — Additional file 2. Timeline of treatment [file 12903_2021_1592_MOESM2_ESM.pptx]

## Slide 1
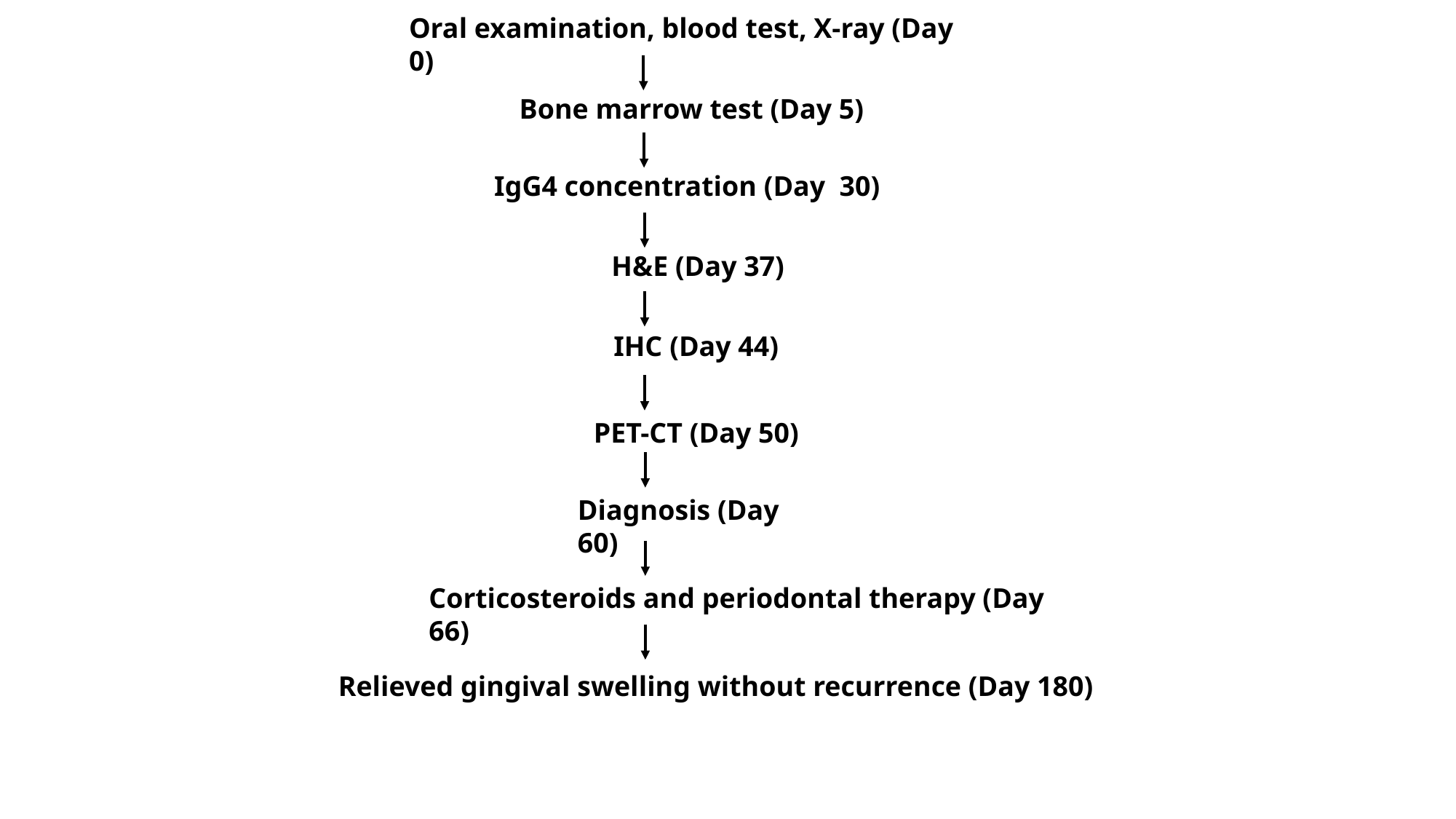

Oral examination, blood test, X-ray (Day 0)
Bone marrow test (Day 5)
 IgG4 concentration (Day 30)
H&E (Day 37)
IHC (Day 44)
PET-CT (Day 50)
Diagnosis (Day 60)
Corticosteroids and periodontal therapy (Day 66)
Relieved gingival swelling without recurrence (Day 180)
